# The Use of ICD-9-CM Coding to Identify COVID-19 Diagnoses and Determine Risk Factors for 30-Day Death Rate in Hospitalized Patients in Italy: Retrospective Study

**DOI:** 10.2196/44062

**Published:** 2024-02-23

**Authors:** Barbara Giordani, Alessandra Burgio, Francesco Grippo, Alessandra Barone, Erica Eugeni, Giovanni Baglio

**Affiliations:** 1 Research, National Outcomes Evaluation Programme (PNE) and International Relations Unit Italian National Agency for Regional Healthcare Services Rome Italy; 2 Italian National Institute of Statistics Rome Italy

**Keywords:** COVID-19, ICD-9-CM coding, hospitalizations, SARS-CoV-2, coronavirus, risk factor, Italy, death rate, monitoring, hospital records, coding, algorithm

## Abstract

**Background:**

In Italy, it has been difficult to accurately quantify hospital admissions of patients with a COVID-19 diagnosis using the Hospital Information System (HIS), mainly due to the heterogeneity of codes used in the hospital discharge records during different waves of the COVID-19 pandemic.

**Objective:**

The objective of this study was to define a specific combination of codes to identify the COVID-19 hospitalizations within the HIS and to investigate the risk factors associated with mortality due to COVID-19 among patients admitted to Italian hospitals in 2020.

**Methods:**

A retrospective study was conducted using the hospital discharge records, provided by more than 1300 public and private Italian hospitals. Inpatient hospitalizations were detected by implementing an algorithm based on specific International Classification of Diseases, Ninth Revision, Clinical Modification (ICD-9-CM) code combinations. Hospitalizations were analyzed by different clinical presentations associated with COVID-19 diagnoses. In addition, 2 multivariable Cox regression models were performed among patients hospitalized “due to COVID-19” from January 1 to December 31, 2020, to investigate potential risk factors associated with 30-day death and the temporal changes over the course of the pandemic; in particular, the 30-day death rates during the first and the second waves were analyzed across 3 main geographical areas (North, Center, and South and Islands) and by discharge wards (ordinary and intensive care).

**Results:**

We identified a total of 325,810 hospitalizations with COVID-19–related diagnosis codes. Among these, 73.4% (n=239,114) were classified as “due to COVID-19,” 14.5% (n=47,416) as “SARS-CoV-2 positive, but not due to COVID-19,” and 12.1% (n=39,280) as “suspected COVID-19” hospitalizations. The cohort of patients hospitalized “due to COVID-19” included 205,048 patients, with a median age of 72 years and a higher prevalence of male patients (n=124,181, 60.6%). The overall 30-day death rate among hospitalized patients due to COVID-19 was 9.9 per 1000 person-days. Mortality was lower for women (hazard ratio [HR]=0.83; *P*<.001) and for patients coming from high migration pressure countries, especially Northern Africans (HR=0.65; *P*<.001) and Central and Eastern Europeans (HR=0.66; *P*<.001), compared to patients coming from Italy and high-income countries. In the southern regions and the Islands, mortality was higher compared to the northern regions (HR=1.17; *P*<.001), especially during the second wave of COVID-19 among patients with a transfer to intensive care units (HR=2.52; *P*<.001).

**Conclusions:**

To our knowledge, the algorithm is the first attempt to define, at a national level, selection criteria for identifying COVID-19 hospitalizations within the HIS. The implemented algorithm will be used to monitor the pandemic over time, and the patients selected in 2020 will be followed up in the next years to assess the long-term effects of COVID-19.

## Introduction

The COVID-19 pandemic affected global health care systems, leading to an increase in hospitalizations and intensive care admissions and a high risk of severe clinical outcomes, particularly in older adults [1].

SARS-CoV-2 infection led to clinical manifestations ranging from asymptomatic or paucisymptomatic forms (medical conditions in which patients experience only a small number of symptoms or mild symptoms) to a wide spectrum of symptoms, such as pneumonia, upper respiratory tract infection, or respiratory failures [2,3].

Some studies showed that the most critically ill patients with COVID-19 in intensive care units (ICUs) required invasive mechanical ventilation and reported a high mortality rate [4]. A meta-analysis of observational studies also found that older age (≥65 years), male gender, and comorbidities (eg, hypertension, cardiovascular diseases, diabetes, chronic obstructive pulmonary diseases, and malignancies) were associated with a greater risk of death from COVID-19 [5]. Although environmental, clinical, and social characteristics have a role as risk factors for SARS-CoV-2 infection and the severity of the disease [6-8], evidence is arising about the importance of human genetics in the onset and course of COVID-19 [9,10].

In Italy, despite early data provided by the COVID-19 Integrated Surveillance System [11], it has been difficult to accurately quantify hospital admissions of patients with a COVID-19 diagnosis using the Hospital Information System (HIS), mainly due to the heterogeneity of codes used in the hospital discharge records (HDRs) during different waves of the COVID-19 pandemic.

Indeed, with the beginning of the COVID-19 pandemic, the World Health Organization provided, in response to member state requests, codes and instructions for COVID-19 coding in the *International Classification of Diseases, 10th Revision* (*ICD-10*) and *International Classification of Diseases, 11th Revision* (*ICD-11*) [12], but not in the *International Classification of Diseases, Ninth Revision, Clinical Modification* (*ICD-9-CM*), which is the international classification of diseases currently used in Italy for coding in the HDRs.

Therefore, on March 20, 2020, the Italian Ministry of Health (MoH) published the first guidelines to ensure the needed homogeneity in the criteria and methods of HDR coding throughout the country [13]. Such guidelines provide instructions to identify COVID-19 and related clinical conditions by adapting unspecific codes already existing in the *ICD-9-CM*. Only with the Ministerial Decree of October 28, 2020 [14], specific *ICD-9-CM* codes were introduced for COVID-19. Annex 1 of the Ministerial Decree and the guidelines issued on February 19, 2021, provided new instructions for both the principal and secondary diagnoses, aiming to overcome differences in coding among different Italian regions. However, the harmonization of COVID-19 information on HDRs has been gradual in different Italian regions, and sometimes, it was done retrospectively by updating and changing codes in HDRs.

The algorithm presented in this paper, based on the COVID-19 codes included in different MoH guidelines, the *ICD-9-CM* SARS codes, and the codes that reported a dramatic increase in 2020, has made it possible to quantify patients with COVID-19–related diagnoses admitted to Italian hospitals. Moreover, the algorithm made it possible to classify hospitalizations as “due to COVID-19,” “SARS-CoV-2 positive, but not due to COVID-19,” and “suspected COVID-19,” and investigate the risk factors associated with mortality “due to COVID-19” among patients admitted to Italian hospitals in 2020.

Moreover, the algorithm is important because other researchers could use it for potential investigations on specific issues related to the pandemic.

## Methods

### Source

A nationwide retrospective study was conducted using the HDRs on health care services in 2020, provided by more than 1300 public and private Italian hospitals. Hospital discharge data are routinely collected by the Italian MoH and contain patient demographic information (eg, gender and age), admission and discharge dates, up to 6 discharge diagnoses (*ICD-9-CM*), up to 11 medical procedures or surgical interventions, and status at discharge (eg, alive, deceased, or transferred to another hospital). In addition, the National Tax Registry was used to determine vital status or death following hospitalization. HDRs were linked with National Tax Registry records using deterministic record linkage.

### Ethical Considerations

This study is conducted in accordance with the Ministerial Decree, “Evaluation in terms of quality, safety and appropriateness of activities provided for accreditation and contractual agreements with health facilities,” released on December 19, 2022, Article 4 [15]. This decree grants a mandate, *inter alia*, to the Italian National Agency for Regional Healthcare Services to monitor hospital activity and publish reports based on anonymized Italian hospitalizations. We did not include any identifiable data of patients’ personal information, including name, identity information, address, and telephone number; therefore, ethics approval was not required.

### Algorithm to Identify COVID-19 Hospitalizations

Inpatient hospitalizations were detected by implementing an algorithm based on specific *ICD-9-CM* code combinations. This was carried out through the integration of 2 distinct approaches. One approach, deductive (ie, from the general rule to practical use), was based on the *ICD-9-CM* codes reported in the official documents of the MoH for COVID-19 case identifications. In addition, we used codes referring to SARS (though not specifically due to SARS-CoV-2), assuming that these codes, mainly at the beginning of the pandemic and before the MoH guidelines publication, were used to indicate COVID-19 hospitalizations. These codes are presented in Table 1.

**Table 1:** Codes from the deductive approach.

Codes sources	Codes
First guidelines by MoH^a^ (March 20, 2020)	078.89 (other specified diseases due to viruses) V01.79 (contact with or exposure to other viral diseases) V07.0 (need for isolation) V71.83 (observation and evaluation for suspected exposure to other biological agents)
MoH Decree (October 28, 2020)	043 (COVID-19 disease) 480.4 (COVID-19 pneumonia) 518.9 (COVID-19 acute respiratory distress syndrome) 519.7 (COVID-19 other respiratory infections) V01.85 (exposure to SARS-CoV-2) V01.82 (exposure to SARS-associated coronavirus) V07.00 (need for isolation after contact with SARS-COV-2) V12.04 (personal history of COVID-19) V71.84 (observation and evaluation for suspected exposure to SARS-COV-2)
Use of *ICD-9-CM*^b^ codes of SARS	079.82 (SARS-associated coronavirus) 480.3 (pneumonia due to SARS-associated coronavirus) V01.82 (exposure to SARS-associated coronavirus)

^a^MoH: Ministry of Health.

^b^ICD-9-CM: International Classification of Diseases, Ninth Revision, Clinical Modification.

The other approach, inductive (ie, from an empirical observation to a general rule) was used to confirm and integrate the algorithm. In detail, comparing the number of hospitalizations between 2019 and 2020, a dramatic increase was found in the principal diagnosis with the *ICD-9-CM* code 484.8 for “pneumonia in other infectious diseases classified elsewhere*.*” This code was presumed to be associated with COVID-19 and was integrated into the algorithm*.*

The hospitalizations were then grouped into clinical profiles according to a hierarchical approach based on 3 main criteria, as follows:

- The main reason for hospitalization: admissions categorized as “due to COVID-19” (codes identifying COVID-19 in the principal diagnosis) or “SARS-CoV-2 positive, but not due to COVID-19” (codes identifying COVID-19 only in secondary diagnosis).
- Degree of certainty: diagnosis confirmed or categorized as “suspected COVID-19”; the latter included hospitalizations with nonspecific diagnoses or V codes (exposure to SARS-CoV-2, observation, or isolation), for which it is difficult to ascertain if they refer to COVID-19 cases.
- Clinical presentation: pneumonia or acute respiratory distress syndrome, other acute respiratory infections, full-blown COVID-19 without respiratory symptoms, and asymptomatic or pauciasymptomatic COVID-19.

All diagnosis codes and their combined use (algorithm) in the principal and secondary diagnoses used to select and classify COVID-19 hospitalizations are shown in Table S1 in Multimedia Appendix 1.

### Statistical Analysis

Hospitalizations were analyzed with respect to the distribution of different clinical presentations associated with diagnoses of COVID-19.

A further analysis was performed only among patients hospitalized “due to COVID-19” from January 1 to December 31, 2020, to investigate potential risk factors associated with 30-day mortality. The cohort included all patients with at least 1 “due to COVID-19” hospitalization, excluding repeated admissions due to transfers to other hospitals or readmissions.

Time-to-event techniques were used to analyze survival. The follow-up of patients began from the hospital admission date until either the ascertainment of death (outcome) or up to 30 days after admission (censoring).

The 30-day death rate was calculated as the total number of all-cause deaths up to 30 days after admission divided by the total follow-up days of hospitalized patients in the cohort and was reported per 1000 patient-days. To investigate the association of one or more risk factors with the 30-day death rate among patients hospitalized “due to COVID-19,” a multivariable Cox proportional-hazards regression model (Cox, 1972) was used, after having checked the proportional hazard assumption.

Sociodemographic data (eg, age, gender, area of residence, or nationality) were considered in the analysis. Patients were classified according to the following geographic areas of nationality: Italy or other high-income countries; migrants from high migration pressure countries (HMPC), which include Central and Eastern European countries (including those belonging to the European Union) and Malta; Northern African countries; other African countries; Central and South American countries, Asian countries (excluding Japan, Israel, and South Korea); and Oceania (excluding Australia and New Zealand) [16].

The transfer to ICU has been used as a proxy for the clinical severity of the disease.

The Charlson Comorbidity Index (CCI) [17,18] was used to account for current comorbidities or comorbidities over the previous 4 years in patients that could affect the death rate. To calculate the CCI, 17 medical conditions were considered [19,20]. The CCI was categorized into the following scores: CCI=0, CCI=1 or 2, and CCI≥3.

To detect differences in the 30-day death rate during the year 2020, three pandemic periods were considered, as follows:

- First wave, including the Italian lockdown (January 1 to May 5, 2020)
- Postlockdown period (May 6 to October 7, 2020)
- Second wave (October 8 to December 31, 2020)

Two further multivariable Cox regression models were performed to assess temporal changes over the course of the pandemic; in particular, the 30-day death rates during the first and second waves were analyzed across 3 main geographical areas (ie, North, Center, and South and Islands) and by discharge wards (ie, ordinary and intensive care).

The selection of patients and hospital admissions was performed using SAS Studio (version 3.81; SAS Institute; Enterprise edition). The Cox proportional-hazards regression was performed using STATA (version 14.0; StataCorp). Tests were 2-sided, and statistical significance was set at *P*<.05.

## Results

### COVID-19 Hospitalizations Analysis

We identified a total of 325,810 hospitalizations with COVID-19-related diagnosis codes within the public and private accredited Italian hospitals throughout the year 2020 (Table 2).

A total of 239,114 (73.4%) out of the initial HDR selection were classified as “due to COVID-19” hospitalizations (in most cases, with a diagnosis of pneumonia and acute respiratory distress syndrome), and 47,416 (14.5%) were classified as “SARS-CoV-2 positive, but not due to COVID-19” hospitalizations, mainly in association with diseases of the circulatory system, injuries, chronic diseases of respiratory system, and complication of pregnancy, childbirth, and the puerperium.

The remaining 12.1% (n=39,280) were “suspected COVID-19 hospitalizations.” A high proportion of such hospitalizations (n=29,327, 9%) were also found in association with other pathological conditions reported in principal diagnosis.

There was a significant increase in the length of ICU stay out of the overall length of hospital stay (calculated in days), compared with the prepandemic period (1,031,037/54,170,360, 1.9% in 2019 vs 1,204,216/46,113,945, 2.6% in 2020; *P*<.001). In addition, this proportion was 2.6% (33,509/1,295,932) in 2020 for the “SARS-CoV-2 positive, but not due to COVID-19” and “suspected COVID-19” hospitalizations, while it increased to 8.4% (281,783/3,355,386) among the “due to COVID-19” hospitalizations (data are not shown in the Tables).

**Table 2:** The number of admissions categorized by reason for hospitalization and clinical presentation of COVID-19 in Italy, 2020.

Reason for hospitalization and clinical presentation		Values (N=325,810), n (%)
**Due to COVID-19, n=239,114 (73.4%)**		
	Pneumonia or acute respiratory distress syndrome	222,655 (93.1)
	Other acute respiratory infections	2275 (1)
	Full-blown COVID-19 (without respiratory symptoms)	5718 (2.4)
	Pauciasymptomatic COVID-19	8466 (3.5)
	Total	239,114 (100)
**SARS-CoV-2–positive but not due to COVID-19 (with secondary diagnoses of COVID-19),** **n=** **47,416 (14.5%)**		
	Diseases of the circulatory system	10,318 (21.8)
	Injuries	6038 (12.7)
	Chronic diseases of the respiratory system	4424 (9.3)
	Complication of pregnancy, childbirth, and the puerperium	4089 (8.6)
	Diseases of the digestive system	3208 (6.8)
	Neoplasms	2993 (6.3)
	Factors influencing health status	2844 (6)
	Infectious and parasitic diseases	2441 (5.1)
	Symptoms, signs, and ill-defined conditions	2328 (4.9)
	Diseases of the genitourinary system	2030 (4.3)
	Diseases of the nervous system and sense organs	1985 (4.2)
	Diseases of the musculoskeletal system and connective tissue	1851 (3.9)
	Mental disorders	1075 (2.3)
	Other	1792 (3.8)
	Total	47,416 (100)
**Due to suspected COVID-19, n=9953 (3.1%)**		
	Pneumonia or acute respiratory distress syndrome	8277 (83.2)
	Other acute respiratory infections in suspected COVID-19	678 (6.8)
	Need for isolation	998 (10)
	Total	9953 (100)
Associated with suspected COVID-19 (non–COVID-19 disease), n=29,237 (9%)		29,327 (100)
Total		325,810 (100)

### “Due to COVID-19” Patients Analysis

Table 3 shows the baseline demographic and clinical characteristics of patients hospitalized “due to COVID-19.”

The cohort included 205,048 patients, with a median age of 72 years. Of these patients, 147,266 (71.8%) were older than 60 years, and there was a higher prevalence of male patients (n=124,181, 60.6%). Additionally, 6.1% (n=12,364) were HMPC citizens coming from Central and Eastern Europe (n=3721, 1.8%), Asia (n=2842, 1.4%), Central and South America (n=2637, 1.3%), Northern Africa (n=1786, 0.9%), and other African countries (n=1378, 0.7%). More than two-thirds of patients resided in the northern Italian regions, while 31.5% (n=64,491) were in Central and Southern Italy. Over the 3 periods of analysis, 39.4% (n=80,843) of patients were hospitalized in the first wave, 53.9% (n=110,446) were hospitalized in the second wave, and only 6.7% (n=13,759) were hospitalized in the postlockdown period. A total of 199,283 (97.2%) patients reported a Charlson Comorbidity Index score lower than or equal to 2.

The overall 30-day death rate in patients hospitalized “due to COVID-19” was 9.9 per 1000 person-days. After adjustment, mortality was lower for women (HR=0.83; *P*<.001) and for patients coming from HMPC. Among the latter, North African patients (HR=0.65; *P*<.001) and patients from Central and Eastern Europe (HR=0.66; *P*<.001) were around 35% less likely to die within 30 days, compared to patients coming from Italy and other high-income countries; Asian patients as well as Central and Southern American patients were 17% less likely to die within 30 days.

**Table 3:** The baseline demographic and clinical characteristics of patients hospitalized “due to COVID-19.”

Characteristics and categories		Patients (N=205,048), n (%)	The 30-day death rate per 1000 person-days (overall=9.9 per 1000)	Adjusted hazard ratio	95% CI
**Gender**					
	Male	124,181 (60.6)	10.2	1	N/A^a^
	Female	80,867 (39.4)	9.4	0.83	0.82-0.85
**Age (years)^b^**					
	0-40	10,164 (5)	0.5	1	N/A
	41-50	15,421 (7.5)	1.1	2.18	1.80-2.63
	51-60	32,197 (15.7)	2.2	3.77	3.17-4.49
	61-70	39,080 (19.1)	5.8	8.99	7.590-10.66
	>70	108,186 (52.7)	17.5	28.98	24.49-34.30
**Nationality**					
	Italy or other high-income countries	192,684 (93.9)	10.5	1	N/A
	North Africa	1786 (0.9)	2.3	0.65	0.54-0.78
	Other African Countries	1378 (0.7)	1.4	0.77	0.59-1.00
	Central and South American countries	2637 (1.3)	2.3	0.83	0.71-0.96
	Asia	2842 (1.4)	1.6	0.83	0.70-0.99
	Central and Eastern Europe	3721 (1.8)	2.5	0.66	0.59-0.75
**Italian geographic area^c^**					
	North	140,557 (68.5)	10.2	1	N/A
	Center	34,195 (16.7)	8.4	0.90	0.88-0.93
	South and Islands	30,296 (14.8)	10.3	1.17	1.14-1.20
**Periods**					
	January 1 to May 5, 2020	80,843 (39.4)	11	1	N/A
	May 6 to October 7, 2020	13,759 (6.7)	4.4	0.45	0.43-0.47
	October 8 to Dec 31, 2020	110,446 (53.9)	9.9	0.85	0.83-0.86
**Charlson Comorbidity Index**					
	0	96,225 (46.9)	6.7	1	
	1 or 2	103,058 (50.3)	12.7	1.37	1.35-1.40
	≥3	5765 (2.8)	19.4	1.68	1.61-1.76
**Ward of discharge**					
	Ordinary care	176,801 (86.2)	8.5	1	N/A
	Intensive care	28,247 (13.8)	19.5	2.55	2.50-2.60

^a^N/A: not applicable.

^b^Median age was 72 years.

^c^The North includes Valle d'Aosta, Piemonte, Liguria, Lombardia, Trentino-Alto Adige, Friuli-Venezia Giulia, Veneto, and Emilia-Romagna regions. The Center includes Toscana, Umbria, Marche, and Lazio regions. The South and Islands include Abruzzo, Molise, Campania, Puglia, Basilicata, Calabria, Sicilia, and Sardegna regions.

In addition, we found a higher 30-day survival in the central regions compared with the northern regions, which were the most affected by COVID-19 (HR=0.90; *P*<.001), while the southern regions and the Islands showed a higher risk of 30-day mortality (HR=1.17; *P*<.001).

Transfers to ICUs involved 13.8% (28,247/205,048) of hospitalized patients.

Patients referred to ICUs were two and a half times more likely to die within 30 days, compared to patients hospitalized in ordinary wards (HR=2.55; *P*<.001).

Table 4 shows the survival analysis by geographic area and wards during 2 pandemic waves.

Over the first wave, among patients in ordinary wards, we found a higher 30-day death rate in the northern regions (10.9 per 1000) compared to the central (7.7 per 1000) and southern islands regions (5.6 per 1000). On the contrary, among patients with a transfer to ICUs, the southern regions and the Islands showed a higher 30-day death rate (28.4 per 1000) compared to the northern regions (15.2 per 1000).

Throughout the second wave, among patients in ordinary wards, there was a significant decrease in 30-day death rates in the northern regions (8.7 per 1000), whereas such rates increased up to 7.4 per 1000 person-days in the southern regions and the Islands. On the other hand, among patients with a transfer to ICUs, the 30-day death rate increased in all geographic areas, particularly in the southern regions and the Islands, where the number of ICU patients more than tripled from the first to the second wave; this resulted in a significantly higher mortality risk in the southern regions and the Islands compared to the northern ones (HR=2.52; *P*<.001).

**Table 4:** Survival analysis of patients in hospitals due to COVID-19 during the first and the second waves of the pandemic by geographic area and wards of discharge in Italy in 2020.

Wards and geographic area		First wave (N=80,843)					Second wave (N=110,446)			
		Patients, n	30-day death rate (per 1000 person-days)	Adjusted hazard ratio^a^	95% CI	Patients, n		30-day death rate (per 1000 person-days)	Adjusted hazard ratio	95% CI
**Ordinary care (first wave: n=68,880; second wave: n=95,915)**										
	North	56,849	10.9	1	N/A^b^	59,416		8.7	1	N/A
	Center	7591	7.7	0.74	0.70-0.78	18,777		7	0.92	0.88-0.95
	South and Islands	4440	5.6	0.65	0.60-0.71	17,722		7.4	1.07	1.03-1.11
**Intensive care unit (first wave: n=11,963; second wave: n=14,531)**										
	North	9144	15.2	1	N/A	7990		17	1	N/A
	Center	1726	15.5	0.92	0.85-1.01	2792		24.5	1.50	1.41-1.60
	South and Islands	1093	28.4	1.66	1.52-1.80	3749		42.5	2.52	2.39-2.65

^a^Adjusted for gender, age class, nationality, and Charlson Comorbidity Index.

^b^N/A: not applicable.

## Discussion

### Principal Findings

In Italian hospitals, due to the lack of COVID-19 codes in the *ICD-9-CM* system, multiple codes were used for COVID-19 patient admissions throughout the pandemic outbreak; this made it difficult to identify COVID-19 hospitalizations. This study enabled the definition of a specific combination of codes to identify COVID-19 hospitalizations at the national level within the HIS. Additionally, it facilitated the investigation of risk factors associated with mortality due to COVID-19 among patients admitted to Italian hospitals in 2020. The selection algorithm made it possible to identify 239,114 hospitalizations “due to COVID-19” from HDR. This number is very similar to the data on hospital admissions gathered from the COVID-19 Integrated Surveillance System, which amounted to 240,542 [11]. The monthly trend (data are not shown in Tables) is also very similar in the 2 data sources. Only in a few regions, the number of cases derived from HDRs is lower than the number obtained from the Surveillance System, especially in the first wave of the pandemic, likely due to a miscoding of data at the beginning of the epidemiological crisis. Nevertheless, the use of HDR data for COVID-19 impact analyses remains preferable to Surveillance System data for several reasons. The Surveillance System is based on data provided by the regions to monitor the evolution of the pandemic with timely data. Nevertheless, HDR data, although less timely, have higher quality, as they are collected following criteria and rules established by national legislation and guidelines, which guarantee greater homogeneity and comparability across different periods and territories. Moreover, HDR contains more information than the Surveillance System, especially on comorbidities, and allows comparisons with non–COVID-19 hospitalizations as well.

Overall, we observed a decrease in the 30-day death rate among patients hospitalized “due to COVID-19” between the 2 waves of the pandemic. This cannot be explained by the introduction of new pharmacological therapies [21]; however, it could be due to a better use of existing therapies associated with gained experience in managing the disease by health care providers and a more effective preparedness of the health care system, including reorganization of hospitals and increasing number of ICU beds for patients with COVID-19 [22].

In particular, in the second wave, mortality was reduced in the northern regions among patients treated in ordinary wards, with a lower proportion of cases referred to ICUs (from 9144/65,993, 13.9.% to 7990/67,406, 11.9%); this may have led to a higher concentration of patients with severe symptoms in ICUs, consistent with a small increase in 30-day death rates (from 15.2 to 17.0 per 1000 person-days). On the contrary, in the southern regions and the Islands, mortality increased between the 2 waves, both in the ordinary wards and in the ICUs, and this might be due to a higher burden on hospital care compared to the first wave, which had affected Southern Italy to a lesser extent. Furthermore, the South reported a decrease in non–COVID-19 hospitalizations, which was greater than the national mean [23], a sign indicating that the hospital system was struggling.

Our study also highlighted the lower 30-day death rate for women compared to men, regardless of age. The observed disparity may be attributed to sex-based differences in immunological responses, comorbidities prevalence, or differences in behaviours [24,25]. Consistent with several studies, this analysis showed that critically ill older patients were at high risk of disease severity and mortality [26,27]. Some studies also highlighted important post–COVID-19 sequelae in patients with cancer, which adversely affect survival and oncological outcomes after recovery [28].

Finally, our findings, consistent with the findings of a study carried out in the city of Milan [29], confirmed that mortality among migrants in hospitals due to COVID-19 was lower than that of Italians and people coming from high-income countries. The lower 30-day death rate observed among HMPC migrants can be explained as the effect of specific selection processes (especially in the early and late stages of the migration path), which tend to maintain the overall health status of the foreign population high. One of them is the so-called “healthy migrant effect,” a dynamic for which only people in good health tend to emigrate [30]. Another selection dynamic, known as the “salmon effect,” is due to the habit of older migrants, especially if ill, to go back to their country of origin [31].

Moreover, an Italian study on data retrieved from the COVID-19 Integrated Surveillance System found an interaction between nationality and the epidemic phases; the analysis stratified by year showed that compared to Italians, mortality and other severe clinical outcomes in non-Italian nationals slightly increased from the last months of 2020 through 2021. The authors hypothesize that reduced access to vaccination by the immigrated population could partly explain such an increase [32], as a high risk of missed vaccination was observed among non–Italian-born people living in Italy, both overall and for individuals ≥50 years of age [33].

### Limitations

This study has several limitations. First, results may be affected by the information quality of the HDR or by missing or incorrect coding of some diagnoses, leading to possible underestimation of comorbidities. Therefore, to minimize the effect of underreporting bias, comorbidities over the previous 4 years were also retrieved.

Second, the study is based on the HDR, which collects administrative data and does not contain clinical information of patients, which is useful to better characterize COVID-19 severity. For this reason, the transfer to the ICU has been used in this study as a proxy for the clinical severity of the disease.

Third, due to the lack of specified COVID-19 codes from the onset of the pandemic, the overall hospitalizations detected through the algorithm might be underestimated.

Finally, the issue of patients hospitalized due to “suspected COVID-19,” which cannot be defined as COVID-19 cases, remains unsolved.

### Conclusions

To our knowledge, the algorithm represents the first attempt to define, at the national level, selection criteria for identifying COVID-19 hospitalizations within the HIS.

While awaiting the adoption of more updated International Classification of Diseases coding systems for hospital diagnoses and procedures in Italy, consistent with the World Health Organization recommendations, the implemented algorithm will be used to monitor the pandemic over time, and the patients selected in 2020 will be followed up in the subsequent years to assess the long-term effects of COVID-19.

Further analyses will be useful to assess the impact of the anti–COVID-19 vaccination campaign on the severity and mortality of the disease and to investigate possible inequalities between population subgroups.

## Appendix

Multimedia Appendix 1 Classification of admissions by reason for hospitalisation and clinical presentation of COVID-19.

## Abbreviations

CCI: Charlson Comorbidity Index
HDR: hospital discharge record
HIS: Hospital Information System
HMPC: high migration pressure countries
HR: hazard ratio
ICD-9-CM: International Classification of Diseases, Ninth Revision, Clinical Modification
ICD-10: International Classification of Diseases, 10th Revision
ICD-11: International Classification of Diseases, 11th Revision
ICU: intensive care unit
MoH: Italian Ministry of Health

## Data availability

The data sets generated and analyzed during this study are available from the corresponding author on reasonable request, by contacting direzione.ricerca@agenas.it.
