# Supplementary material for: The Use of ICD-9-CM Coding to Identify COVID-19 Diagnoses and Determine Risk Factors for 30-Day Death Rate in Hospitalized Patients in Italy: Retrospective Study
Source: JMIR Public Health Surveill. 2024 Feb 23;10:e44062. doi: 10.2196/44062 (PMC10906716; doi:10.2196/44062)
Supplement: Multimedia Appendix 1 [file publichealth_v10i1e44062_app1.docx]

| Appendix 1 | | | |
| --- | --- | --- | --- |
| **Table S1 - Classification of admissions by reason for hospitalisation and clinical presentation of COVID-19** | | | |
| **Reason for hospitalisation** | **Clinical presentation** | **ICD-9-CM codes** | |
|  |  | **Principal diagnosis** | **Secondary diagnoses** |
| **Due to COVID-19** | **Pneumonia/ARDS** | Pneumonia/ARDS due to COVID-19: '48041', '48042', '51891', '51892', '4803' | - |
|  |  | Pneumonia/ARDS: '4808', '4809', '4848', '485', '486', '51881', '51882', '5190*' | COVID-19: '0431', '04311', '04312', '0432', '04321', '04322', '0433', '04331', '04332', '48041', '48042', '51891', '51892', '4803', '51971', '51972’, '07889', '07982' |
|  |  | COVID-19 (except pneumonia/ARDS): '0431', '04311', '04312', '0432', '04321', '04322', '0433', '04331', '04332', '51971', '51972’, '07889', '07982' | Pneumonia/ARDS due to COVID-19: '48041', '48042', '51891', '51892', '4803' |
|  |  | V codes COVID-19 associated: 'V0185', 'V0179', 'V7183’,'V070’, 'V7184’, 'V0700’, 'V1204’, 'V0182’ |  |
|  |  | Acute respiratory infections: '460', '461', '462', '463', '464', '465', '466, '490', '5110', '5118', '5119', '51911' |  |
|  |  | COVID-19 (except pneumonia/ARDS): '0431', '04311', '04312', '0432', '04321', '04322', '0433', '04331', '04332', '51971', '51972’, '07889', '07982' | Pneumonia/ARDS: '4808', '4809', '4848', '485', '486', '51881', '51882', '5190*' |
|  | **Other acute respiratory infections** | Acute respiratory infections (except pneumonia/ARDS) due to COVID-19: '51971', '51972' | - |
|  |  | Acute respiratory infections (except pneumonia/ARDS): '460', '461', '462', '463', '464', '465', '466, '490', '5110', '5118', '5119', '51911' | COVID-19 (except pneumonia/ARDS): '0431', '04311', '04312', '0432', '04321', '04322', '0433', '04331', '04332', '51971', '51972’, '07889', '07982' |
|  |  | COVID-19 (except acute respiratory infections): '0431', '04311', '04312', '0432', '04321', '04322', '0433', '04331', '04332', '07889', '07982' | Acute respiratory infections (except pneumonia/ARDS) due to COVID-19: '51971', '51972' |
|  |  | V codes COVID-19 associated: 'V0185', 'V0179', 'V7183’,'V070’, 'V7184’, 'V0700’, 'V1204’, 'V0182’ |  |
|  |  | COVID-19 (except acute respiratory infections): '0431', '04311', '04312', '0432', '04321', '04322', '0433', '04331', '04332', '07889', '07982' | Acute respiratory infections (except pneumonia/ARDS): '460', '461', '462', '463', '464', '465', '466, '490', '5110', '5118', '5119', '51911' |
|  | **Full-blown COVID-19 (w/o respiratory symptoms)** | Full-blown COVID-19: '0431', '04311', '04312' | - |
|  |  | V codes COVID-19 associated: 'V0185', 'V0179', 'V7183’,'V070’, 'V7184’, 'V0700’, 'V1204’, 'V0182’ | Full-blown COVID-19: '0431', '04311', '04312' |
|  | **Pauci-asymptomatic COVID-19** | Pauci-asymptomatic COVID-19: '0432', '04321', '04322', '0433', '04331', '04332'; Other diseases due to viruses: '07889'; SARS-associated coronavirus: '07982' | - |
|  |  | V codes COVID-19 associated: 'V0185', 'V0179', 'V7183’,'V070’, 'V7184’, 'V0700’, 'V1204’, 'V0182’ | Pauci-asymptomatic COVID-19: '0432', '04321', '04322', '0433', '04331', '04332'; Other diseases due to viruses: '07889'; SARS-associated coronavirus: '07982' |
| **SARS-CoV-2 positive but not due to COVID-19** | **Non-COVID-19 disease (with secondary diagnoses of COVID-19)** | Non-COVID-19 (except acute respiratory infections) | COVID-19: '0431', '04311', '04312', '0432', '04321', '04322', '0433', '04331', '04332', '48041', '48042', '51891', '51892', '4803', '51971', '51972’, '07889', '07982' |
| **Due to suspected COVID-19** | **Pneumonia/ARDS in suspected COVID-19** | Pneumonia in other infectious diseases classified elsewhere: '4848' | - |
|  |  | Pneumonia/ARDS: '4808', '4809', '485', '486', '51881', '51882', '5190*' | V codes COVID-19 associated: 'V0185', 'V0179', 'V7183’,'V070’, 'V7184’, 'V0700’, 'V1204’, 'V0182’ |
|  |  | V codes COVID-19 associated: 'V0185', 'V0179', 'V7183’,'V070’, 'V7184’, 'V0700’, 'V1204’, 'V0182’ | Pneumonia/ARDS: '4808', '4809', '4848', '485', '486', '51881', '51882', '5190*' |
|  | **Other acute respiratory infections in suspected COVID-19** | Acute respiratory infections (except pneumonia/ARDS): '460', '461', '462', '463', '464', '465', '466, '490', '5110', '5118', '5119', '5191' | V codes COVID-19 associated: 'V0185', 'V0179', 'V7183’,'V070’, 'V7184’, 'V0700’, 'V1204’, 'V0182’ |
|  |  | V codes COVID-19 associated: 'V0185', 'V0179', 'V7183’,'V070’, 'V7184’, 'V0700’, 'V1204’, 'V0182’ | Acute respiratory infections (except pneumonia/ARDS): '460', '461', '462', '463', '464', '465', '466, '490', '5110', '5118', '5119', '51911' |
|  | **Need for isolation** | V codes COVID-19 associated: 'V0185', 'V0179', 'V7183’,'V070’, 'V7184’, 'V0700’, 'V1204’, 'V0182’ | Non-COVID-19 (except acute respiratory infections) |
| **Associated with suspected COVID-19** | **Non-COVID-19 disease (with secondary diagnoses of suspected COVID-19)** | Non-COVID-19 (except acute respiratory infections) | V codes COVID-19 associated: 'V0185', 'V0179', 'V7183’,'V070’, 'V7184’, 'V0700’, 'V1204’, 'V0182’ |
|  |  |  | Pneumonia in other infectious diseases classified elsewhere: '4848' |
| The sign * means "*any codes"* | | | |
